# The role of p63 in embryonic external genitalia outgrowth in mice

**DOI:** 10.1111/dgd.12840

**Published:** 2023-02-02

**Authors:** Kosei Tanaka, Daisuke Matsumaru, Kentaro Suzuki, Gen Yamada, Shinichi Miyagawa

**Affiliations:** ^1^ Department of Biological Science and Technology, Faculty of Advances Engineering Tokyo University of Science Katsushika Japan; ^2^ Laboratory of Hygienic Chemistry and Molecular Toxicology Gifu Pharmaceutical University Gifu Japan; ^3^ Faculty of Life and Environmental Sciences University of Yamanashi Yamanashi Japan; ^4^ Institute of Advanced Medicine Wakayama Medical University Wakayama Japan; ^5^ Division of Biological Environment Innovation, Research Institute for Science and Technology Tokyo University of Science Katsushika Japan

**Keywords:** cloaca, external genitalia, Fgf8, hedgehog, p63, Wnt

## Abstract

Embryonic external genitalia (genital tubercle [GT]) protrude from the cloaca and outgrow as cloacal development progresses. Individual gene functions and knockout phenotypes in GT development have been extensively analyzed; however, the interactions between these genes are not fully understood. In this study, we investigated the role of p63, focusing on its interaction with the Shh–Wnt/Ctnnb1–Fgf8 pathway, a signaling network that is known to play a role in GT outgrowth. p63 was expressed in the epithelial tissues of the GT at E11.5, and the distal tip of the GT predominantly expressed the ΔNp63α isoform. The GTs in *p63* knockout embryos had normal *Shh* expression, but CTNNB1 protein and *Fgf8* gene expression in the distal urethral epithelium was decreased or lost. Constitutive expression of CTNNB1 in *p63*‐null embryos restored *Fgf8* expression, accompanied by small bud structure development; however, such bud structures could not be maintained by E13.5, at which point mutant GTs exhibited severe abnormalities showing a split shape with a hemorrhagic cloaca. Therefore, p63 is a key component of the signaling pathway that triggers *Fgf8* expression in the distal urethral epithelium and contributes to GT outgrowth by ensuring the structural integrity of the cloacal epithelia. Altogether, we propose that p63 plays an essential role in the signaling network for the development of external genitalia.

## Graphical abstract

We investigated the role of p63 during embryonic external genitalia development. We found that p63 is a key component of the signaling pathway that triggers Fgf8 expression in the distal urethral epithelium and contributes to embryonic external genitalia outgrowth.

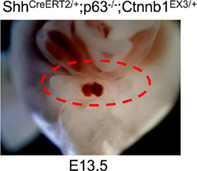

## 1 INTRODUCTION

The genital tubercle (GT) is a primordium of the external genitalia that develops as an appendage of the body trunk during early development, similar to limb buds. In mice, the GT develops from the cloacal region as genital swellings on embryonic day (E) 10.5, and visibly outgrows as a consequence of mesenchymal swelling around the cloaca at E11.5 (Haraguchi et al., 2000, 2001; Perriton et al., 2002). GT outgrowth is accompanied by cloacal development and urethral plate epithelium (future urethra) formation on the ventral side. This outgrowth and early patterning of the GT is commonly observed in both males and females, independent of androgen action (Amato & Yao, 2021; Matsushita et al., 2018; Miyagawa et al., 2011; Miyagawa, Satoh, et al., 2009).

Appendage development, including that of the GT and limb buds, is a representative model for analyzing the reciprocal interactions of various developmental regulatory genes. Outgrowing embryonic buds are often composed of a unique cell population in the distal epithelium, accompanied by adjacent proliferating mesenchyme, which eventually gives rise to a bud structure. During limb development, the apical ectodermal ridge (AER), a specialized epithelium at the distal tip of the limb bud, regulates the establishment and maintenance of outgrowth (Johnson & Tabin, 1997; Tickle, 1995). As a source of signals essential for appendage development, the AER expresses FGF8 (and other FGF ligands) to supply cells for the expansion of mesenchymal tissue and patterning of limb formation (Crossley et al., 1996; Sun et al., 2002; Vogel et al., 1996). Likewise, temporal signaling centers controlling appendage development are also formed in the GT, namely, the distal urethral epithelium (DUE) at the tip of the cloacal membrane (Haraguchi et al., 2000, 2001; Lin et al., 2008; Ogino et al., 2001). Since the discovery of *Fgf8* expression in the DUE and its mitogenic activity in the GT mesenchyme (Haraguchi et al., 2000), regulation of *Fgf8* expression has become a major research topic in GT development. Shortly thereafter, hedgehog signaling was identified as playing a key role in *Fgf8* expression and GT protrusion. *Shh* knockout (KO) embryos do not express *Fgf8* in the DUE and show GT agenesis and persistent cloaca (Haraguchi et al., 2001; Mo et al., 2001). Furthermore, studies of reciprocal gene regulation using mutant mice revealed that Wnt/Ctnnb1 is a key signal for the interplay between hedgehog signaling and *Fgf8* expression (Lin et al., 2009; Miyagawa, Moon, et al., 2009).

In addition to Shh–Wnt/Ctnnb1–Fgf8 pathway‐related genes, various KO mouse studies have significantly advanced our knowledge of individual gene functions in GT outgrowth, including the roles of bone morphogenetic proteins (BMPs) (Lin et al., 2009, Miyagawa, Moon, et al., 2009), non‐canonical Wnt signaling (Alcantara et al., 2021; Oishi et al., 2003; Seifert et al., 2009; Suzuki et al., 2003), retinoic acid signaling (Liu et al., 2012; Ogino et al., 2001), Hox genes (Dollé et al., 1991; Morgan et al., 2003; Warot et al., 1997), and transformation‐related protein 63 (encoded by *TP63* [human]/*Trp63* [mouse]; hereafter referred to as p63) (Ince et al., 2002; Suzuki et al., 2008). p63, a member of the tumor suppressor p53 protein family, plays important roles in multiple developmental processes, and a pivotal role in epithelial homeostasis in particular. Notably, *p63* KO mice exhibit striking defects in the embryonic epidermis and epidermal appendages and show rudimentary or absent limbs and GTs (Ince et al., 2002; Mills et al., 1999; Suzuki et al., 2008; Yang et al., 1999). In *p63* KO limb buds, the AER structure is not discernible, showing a single‐layered epithelium at the distal tip, and *Fgf8* expression is progressively reduced (Koster et al., 2007; Mills et al., 1999; Su et al., 2009; Yang et al., 1999). Therefore, maintenance of the structural integrity of the AER by p63 is necessary for limb protrusion.

Alternate promoter usage was employed to generate two N‐terminal variants of p63, TAp63 and ΔNp63 (Yang et al., 1998). TAp63 contains a transactivation (TA) domain in the N‐terminus, whereas ΔNp63 is N‐terminally truncated; however, the ΔNp63 isoform is still transcriptionally active because of the presence of another transactivation domain (TA2) between the oligomerization and sterile alpha motif (SAM) domains (Ghioni et al., 2002). Furthermore, both N‐terminal isoforms give rise to different C‐terminal variants (mainly α, β, and γ) through alternative splicing; thus, six major isoforms have been identified. The SAM domain, an important protein–protein interaction domain located in the C‐terminus, is present only in the α isoform.

Analyses of KO mice have revealed the functions of individual genes in GT development (Hashimoto et al., 2019; Yamada et al., 2003; Yamada et al., 2006); however, the integrated signaling cascade remains elusive. *p63* KO embryos fail to express *Fgf8* in the DUE and exhibit defective GT protrusion (Ince et al., 2002; Suzuki et al., 2008). However, the interaction between p63 and the Shh–Wnt/Ctnnb1–Fgf8 cascade remains unclear. In the current study, we aimed to investigate the relationship between p63 and the Shh–Wnt/Ctnnb1 pathway, focusing on the regulation of *Fgf8* expression as a DUE marker. We demonstrated that p63 can regulate CTNNB1 and *Fgf8* expression in the DUE. Constitutive expression of CTNNB1 in the *p63*‐null background could lead to the formation of a bud structure in the cloacal region at E11.5, but it collapsed mid‐structure by E13.5. Hence, p63 cooperatively regulates *Fgf8* expression with hedgehog and Wnt/Ctnnb1 signals and helps to maintain epithelial integrity of the cloaca, which is indispensable for appendage outgrowth.

## 2 MATERIALS AND METHODS

### 2.1 Animals

C57BL/6J (Sankyo, Tokyo, Japan), *p63* (Mills et al., 1999), *Shh* (Chiang et al., 1996), *Shh* ^*CreERT2*^ (Harfe et al., 2004), *Ctnnb1* ^*EX3*^ (Harada et al., 1999), and *Ctnnb1* ^*flox*^ (Huelsken et al., 2001) mice were maintained under a 12 h light/12 h dark cycle at 23–25°C. The day on which a vaginal plug was detected was designated E0.5. Embryos were collected from at least three pregnant females for each experiment. All procedures and protocols were approved by the Institutional Animal Care and Use Committee of Tokyo University of Science. To obtain *Shh* ^*CreERT2/+*^ *;p63* ^*−/−*^ *;Ctnnb1* ^*EX3/+*^ embryos, *Shh* ^*CreERT2/+*^ *;p63* ^*+/−*^ *;Ctnnb1* ^*EX3/EX3*^ male were crossed with *p63* ^*+/−*^ females, and *Shh* ^*+/+*^ *;p63* ^*+/+*^ *;Ctnnb1* ^*EX3/+*^ siblings were used as controls. Tamoxifen (Sigma‐Aldrich, St. Louis, MO, USA) was dissolved in sesame oil (Kanto Chemical Co., Inc., Tokyo, Japan) and injected into pregnant mice (2 mg/40 g body weight). Under these conditions, no overt teratologic effects on the urogenital organs have been observed (Miyagawa, Moon, et al., 2009) and there was no leakage of Cre activity in the mock (no tamoxifen) control in the *Shh* ^*CreERT2*^ line (Harfe et al., 2004; Seifert et al., 2010).

### 2.2 Histology

Mouse embryos were fixed overnight in 4% paraformaldehyde (Sigma‐Aldrich) in phosphate‐buffered saline and dehydrated with methanol. Eight micrometer‐thick serial sections were prepared after embedding in paraffin. Hematoxylin and eosin staining, immunohistochemistry, and in situ hybridization for gene expression analysis were performed as previously described (Miyagawa & Iguchi, 2015; Miyagawa, Moon, et al., 2009). Immunohistochemistry was performed using anti‐CTNNB1 (610154) (BD Biosciences, Franklin Lakes, NJ, USA), anti‐pan‐p63 (4A4, sc‐8431), anti‐ΔNp63 (sc‐8609), and anti‐TAp63 (sc‐8608) (Santa Cruz Biotechnology, Inc., Santa Cruz, CA, USA) antibodies. Immunofluorescence signals were detected using Alexa Fluor‐conjugated secondary antibodies (Thermo Fisher Scientific, Waltham, MA, USA) and counterstained with Hoechst 33342 (Sigma‐Aldrich). Riboprobe templates for in situ hybridization were kindly provided by Dr. B. Hogan (*Fgf8*) and Dr. C. Shukunami (*Shh*).

### 2.3 Plasmid DNA and luciferase assay

A conserved region at the 3′ end of the *Fgf8* locus has been previously described (Beermann et al., 2006; Miyagawa, Moon, et al., 2009). DNA fragments of conserved region 3 (CR3) of the murine *Fgf8* locus, obtained from the BAC clone RPCI23‐98F2 by PCR, were inserted into the pGL4.24 vector (Promega Corporation, Madison, WI, USA). Mouse *p63* genes were constructed by standard RT‐PCR procedures and were subcloned into the pcDNA3.1 mammalian expression vector (Thermo Fisher Scientific). The mouse *Ctnnb1* expression vector was kindly provided by Dr. S. Kume (Takahashi et al., 2000).

HaCaT cells were maintained in Dulbecco's Modified Eagle Medium supplemented with 10% FBS. Cells were transfected with expression and reporter plasmids using FuGENE HD (Promega Corporation), according to the manufacturer's instructions. At 24–30 h post‐transfection, luciferase activity was measured by chemiluminescence using the Dual‐Luciferase Reporter Assay System (Promega Corporation). Values were normalized to Renilla luciferase activity. At least three independent experiments were performed. Statistical analysis was performed by analysis of variance followed by the Tukey–Kramer test; differences with *p* < .05 were considered significant. Error bars represent SEM.

### 2.4 Immunoblotting

HaCaT cells were transfected with ΔNp63α, ΔNp63β, and ΔNp63γ expression vectors as described above. Transfected cells and embryonic GTs were homogenized with Laemmli sample buffer, followed by SDS‐PAGE and transfer to nitrocellulose membranes. Immunoblotting was performed with an anti‐p63 antibody (4A4), and the signal was detected using an ECL kit (Thermo Fisher Scientific).

### 2.5 Chromatin immunoprecipitation assay

Chromatin immunoprecipitation (ChIP) assays were performed using a ChIP assay kit (Upstate Biotechnology, Inc., Lake Placid, NY, USA). The distal GT region containing the DUE was dissected from embryos at E12.5. Two micrograms of anti‐p63 antibody (4A4) was used. Normal rabbit or mouse immunoglobulin (Dako Agilent, Carpinteria, CA, USA) was used as the mock control. More than three independent experiments were performed. PCR was performed using the following primers: *CR1*, CAG AGA GAG CCG TTT GTG TTG G and TCA AAG CCC CGT AAT TAC AAT TGC; *CR3*, CTG GCT GAA AGC CAC AGA CG and GCT GGG TCT CTG CTG GTA ACC.

## 3 RESULTS

### 3.1 ΔNp63α is a major isoform in the outgrowing GT

**FIGURE 1:**
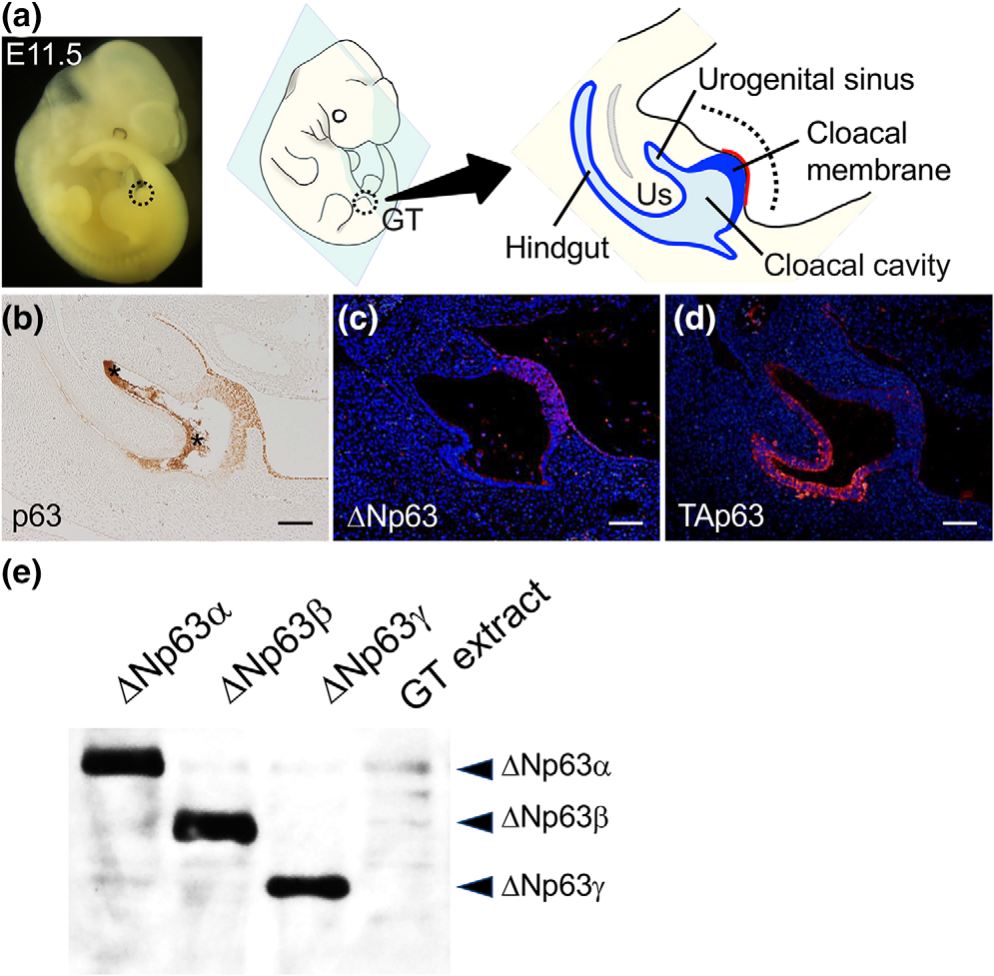
p63 protein expression in developing mouse genital tubercles (GTs) at embryonic day (E) 11.5. (a) A picture of a murine embryo and a schematic illustration of the outgrowing GT at E11.5. The dotted line indicates the GT. Blue regions indicate endoderm‐derived epithelia. The tip of the GT includes the thick cloacal membrane, which comprises the distal urethral epithelium (DUE) (see Figure 2e for *Fgf8* expression in the DUE). The red line indicates adjacent ectoderm‐derived epithelium (GT ectoderm). (b–d) Immunostaining with anti‐pan‐p63 (b), anti‐ΔNp63 (c), and TAp63 (d) antibodies in the GTs at E11.5. Pan‐p63 is expressed in the endodermal and ectodermal epithelia. The ΔNp63 isoform is specifically expressed in the cloacal membrane and adjacent ectoderm. Asterisks indicate background signals. (e) Immunoblotting with anti‐pan‐p63 in the lysate of HaCaT cells overexpressing ΔNp63α, ΔNp63β, and ΔNp63γ as controls and of the murine GT at E11.5.

The murine GT visibly protrudes as a bud structure in the cloacal region at E10.5–E11.5. The GT is composed of endodermal epithelium (cloacal membrane, urogenital sinus, and hindgut), surrounding mesenchyme, and adjacent ectodermal epithelium overlaying the GT surface (hereafter referred to as GT ectoderm) (Figure 1a). We first confirmed p63 expression in the GT at E11.5 (Cheng et al., 2006; Suzuki et al., 2008). Pan‐p63 immunoreactivity was observed in the epithelial tissues of the GT, with relatively high signals in the cloacal membrane and GT ectoderm (Figure 1b). Next, the distinct N‐terminal isoforms of p63 were examined using antibodies specific for either TAp63 or ΔNp63. ΔNp63 was specifically expressed in the cloacal membrane and GT ectoderm, whereas TAp63 was expressed in the endoderm of the urogenital sinus, urorectal septum, and hindgut (Figure 1c, d). We then performed p63 immunoblotting analysis using GT lysate and lysate from murine ΔNp63α‐, ΔNp63β‐, or ΔNp63γ‐overexpressing cells, and determined that ΔNp63α is the predominant isoform expressed in the GT at E11.5 (Figure 1e). The ΔNp63β isoform was also expressed but at a much lower level than ΔNp63α.

### 3.2 Expression pattern of regulatory genes for GT outgrowth

**FIGURE 2:**
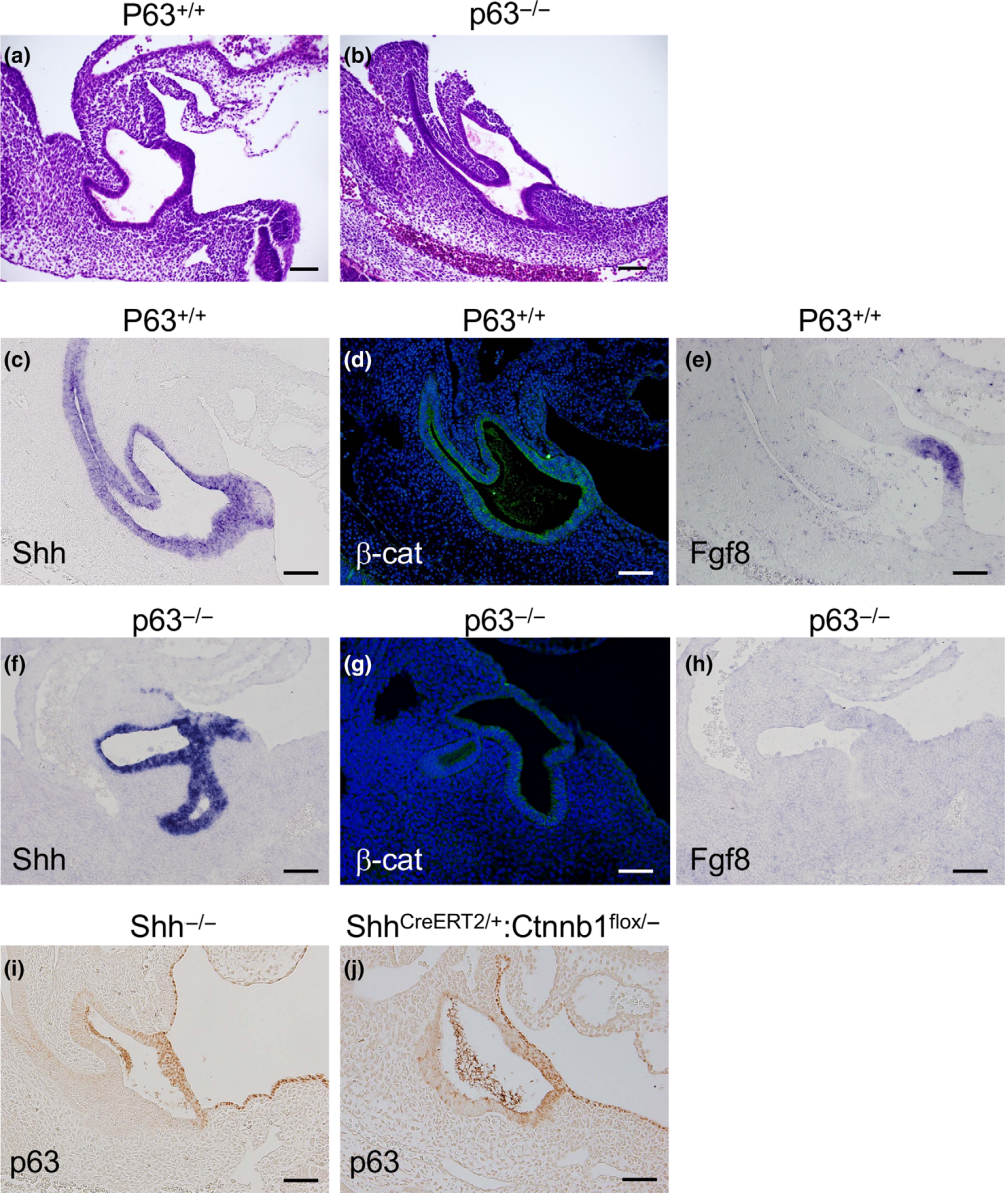
Gene expression pattern of genital tubercles (GTs) at E11.5. (a, b) Control (a) and *p63* KO (b) embryos without GT protrusion. (c–h) *Shh* (c, f), CTNNB1 (d, g), and *Fgf8* (e, h) expression in control (c–e) and *p63* mutant GTs (f–h). CTNNB1 and *Fgf8* expression is decreased or lost in *p63* KO embryos (g, h). (i, j) p63 is expressed in the distal GT of *Shh* KO (i) and endoderm‐specific *Ctnnb1* conditional KO mice (j). Scale bars: 100 μm.

GT outgrowth was defective in *p63* KO embryos, and the cloacal membrane and GT ectoderm were poorly developed (Figure 2a, b). To investigate the interaction with the Shh–Wnt/Ctnnb1–Fgf8 pathway, a key signaling pathway for GT outgrowth, we evaluated gene expression patterns in the *p63* KO GTs at E11.5. In control mice, *Shh* gene and CTNNB1 protein expression were observed in endoderm‐derived epithelium (Figure 2c, d). CTNNB1 was found to be expressed in the GT ectoderm (Figure 2d). *Fgf8* expression was confined to the DUE (Figure 2e). In *p63* KO embryos, *Shh* was expressed, but overall CTNNB1 expression was decreased compared to that in controls (Figure 2f, g). *Fgf8* expression was not observed in *p63* KO GTs (Figure 2h).

We then assessed p63 expression in the GT of *Shh* KO and *Ctnnb1* conditional KO mice (*Shh* ^*CreERT2/+*^ *;Ctnnb1* ^*flox/−*^). Similar to *p63* KO mice, *Shh* KO mice showed GT agenesis accompanied by a poorly developed cloacal membrane and GT ectoderm, but p63 was still expressed in such epithelia (Figure 2i). Wnt/Ctnnb1 signaling is required for caudal body formation during early embryonic development (Dunty Jr. et al., 2008; Takada et al., 1994). To focus on the specific role of CTNNB1 in GT outgrowth, we employed the temporally inducible *Shh* ^*CreERT2*^ line, which is suitable for analyzing gene function during GT outgrowth (Miyagawa, Moon, et al., 2009). *Shh* ^*CreER/+*^ *;Ctnnb1* ^*flox/−*^ embryos treated with tamoxifen at E9.5 expressed p63 in the GTs (Figure 2j). In summary, p63 expression is independent of either hedgehog or Wnt/Ctnnb1 signaling at this stage.

### 3.3 Possible regulation of *Fgf8* expression by p63

**FIGURE 3:**
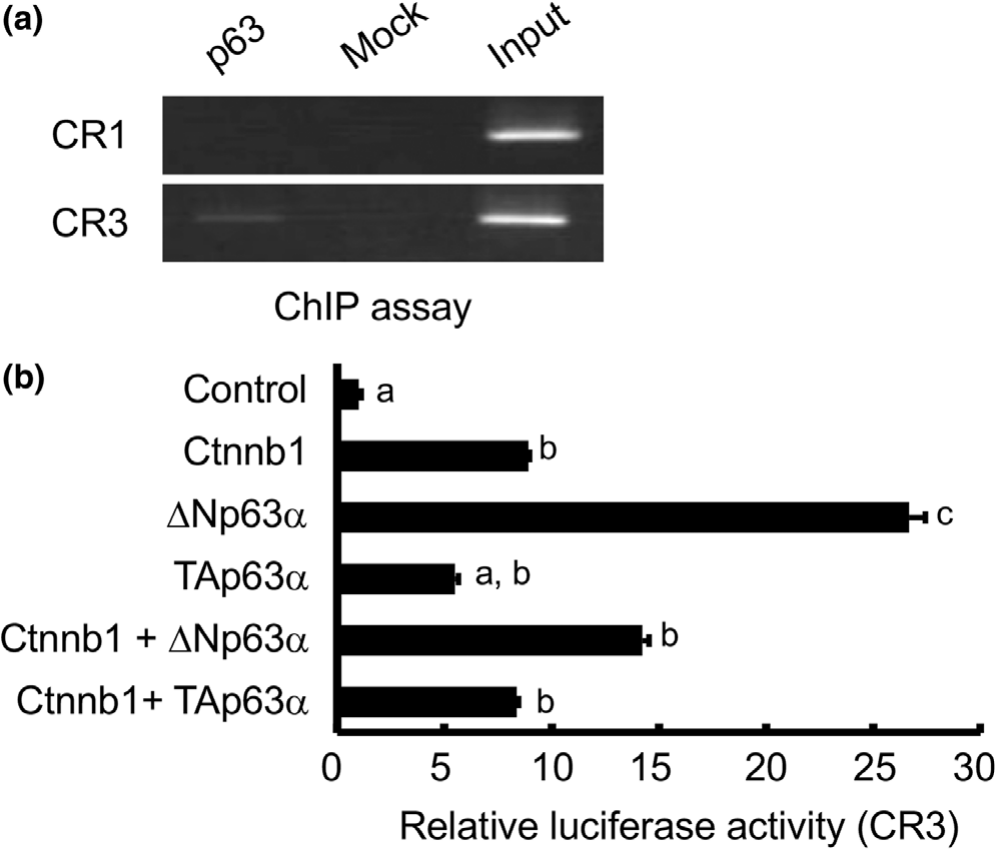
Regulation of *Fgf8* expression by p63. (a) ChIP‐PCR assay on the genital tubercle (GT) reveals that p63 can bind to CR3 but not CR1 of the *Fgf8* gene locus. (b) The CR3 enhancer activates expression of a luciferase reporter in response to ΔNp63α overexpression. Different letters designate significant differences among groups as determined by the Tukey–Kramer test after two‐way analysis of variance. Error bars represent the SEM.

We previously showed that CTNNB1 directly regulates *Fgf8* expression in the DUE (Miyagawa, Moon, et al., 2009). The current expression analysis thus suggests that p63 regulates *Fgf8* expression by controlling CTNNB1 function in the cloacal membrane. It is also possible that *Fgf8* expression is regulated by p63. To investigate this possibility, we performed a ChIP assay followed by PCR using primers designed to amplify a region at the 3′ end of the *Fgf8* locus, CR3, a candidate enhancer that regulates *Fgf8* expression in the AER and DUE (Beermann et al., 2006; Miyagawa, Moon, et al., 2009). p63‐specific enrichment was observed in the extracts of the GTs (Figure 3a). PCR amplification of the 5′ flanking region of *Fgf8*, which is also highly conserved in vertebrates, yielded no enrichment when used as a negative control. The CR3 enhancer/luciferase reporter was activated by Ctnnb1 overexpression in HaCaT cells (Figure 3b) (Miyagawa, Moon, et al., 2009). ΔNp63α also activates the CR3 reporter gene, whereas the TAp63α isoform does not. We then co‐expressed CTNNB1 and either of the p63α isoforms. Neither ΔNp63α nor TAp63α increased the CTNNB1‐induced reporter activity, suggesting no additive and/or synergistic effects between CTNNB1 and the p63α isoforms.

### 3.4 Effects of constitutively expressed CTNNB1 on GT outgrowth in p63 KO GTs

**FIGURE 4:**
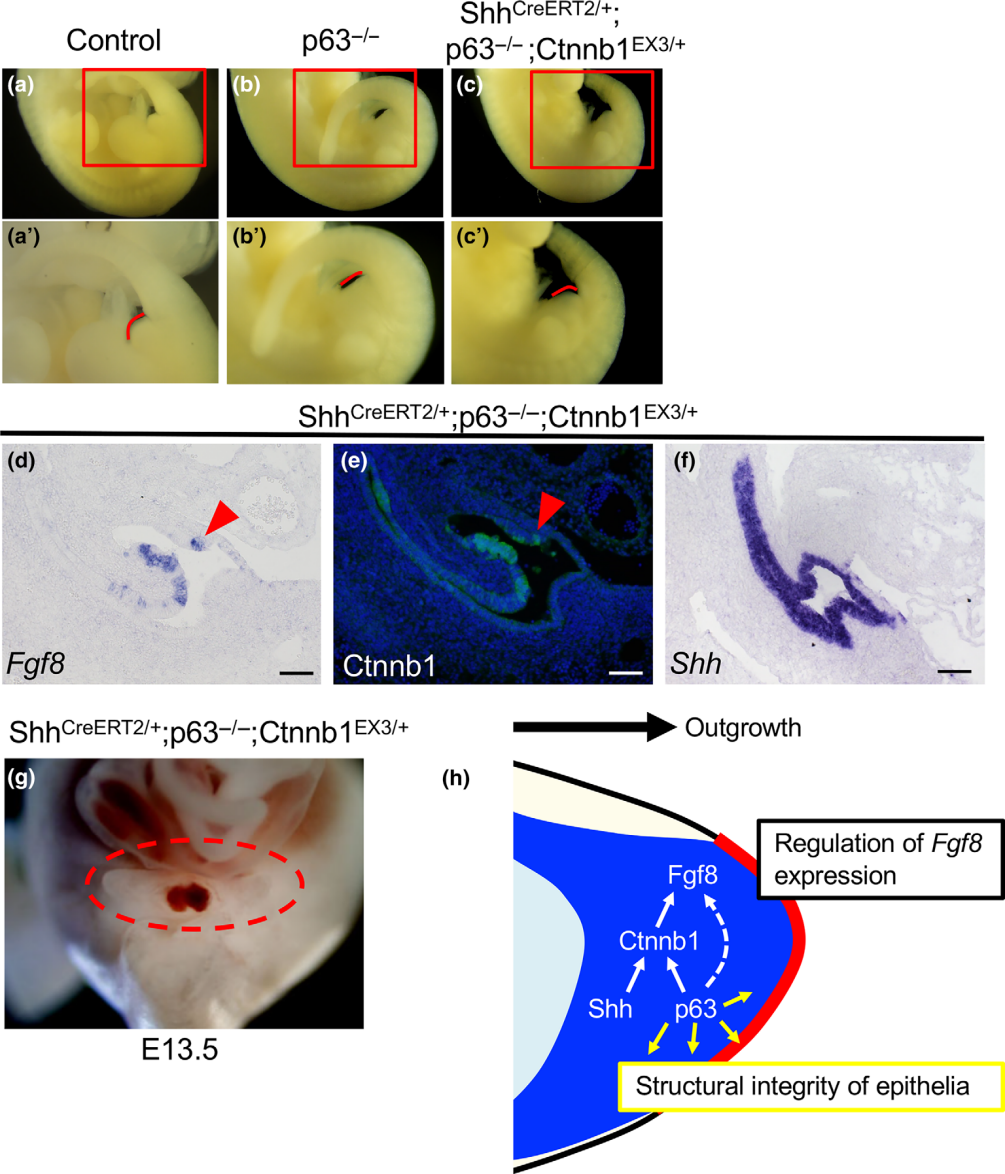
Partial rescue of genital tubercle (GT) outgrowth in *p63* mutants by constitutive expression of CTNNB1. (a, b) Control GT (a) and *p63* KO embryos with defective GT (b) at E11.5. (c) *Shh* ^*CreERT2/−*^ *;p63* ^*−/−*^ *;Ctnnb1* ^*EX3/+*^ embryos exhibit a small bud structure (red curve) at E11.5. (a'–c′) Enlarged views within the frames of (a–c). (d–f) Expression of *Fgf8* (d), CTNNB1 (e), and *Shh* (f) in the GTs of *Shh* ^*CreERT2/−*^ *;p63* ^*−/−*^ *;Ctnnb1* ^*EX3/+*^ embryos at E11.5. *Fgf8* expression overlaps with regions of augmented CTNNB1 expression (d, e). Red arrowheads indicate *Fgf8* and CTNNB1 expression in the distal GT region. (g) The GTs of *Shh* ^*CreERT2/−*^ *;p63* ^*−/−*^ *;Ctnnb1* ^*EX3/+*^ embryos show lateral swelling, but fail to develop a mid‐region, resulting in a split shape with a hemorrhagic cloaca. The dotted circle indicates a GT‐like structure or prospective GT region. (h) Proposed signaling cascade controlling GT outgrowth in mice. p63 and Shh–Wnt/Ctnnb1 comprehensively regulate *Fgf8* expression. p63 plays a role in the maintenance of epithelial cell structure and integrity. Blue regions indicate endoderm‐derived epithelia and the red line indicates GT ectoderm.

Constitutive expression of CTNNB1 can partially rescue *Fgf8* expression and GT outgrowth in a *Shh*‐null background (Miyagawa, Moon, et al., 2009). Thus, we investigated whether constitutive expression of CTNNB1 could also rescue the phenotypes of *p63* KO GTs. We generated *Shh* ^*CreERT2/+*^ *;p63* ^*−/−*^ *;Ctnnb1* ^*EX3/+*^ mutant embryos, which constitutively express CTNNB1 (CTNNB1^EX3^) in the endodermal epithelia, including the cloacal membrane, in a *p63*‐null background. Tamoxifen treatment at E9.5 resulted in visible GT protrusion at E11.5 (Figure 4a–c). *Fgf8* was expressed at a high level in the cloacal epithelium of *Shh* ^*CreERT2/+*^ *;p63* ^*−/−*^ *;Ctnnb1* ^*EX3/+*^ embryos, and the *Fgf8* expression domain coincided with the location of CTNNB1 augmentation (Figure 4d, e). *Shh* expression levels were not affected despite the heterozygous background of *Shh* (Figure 4f). Thus, constitutive expression of CTNNB1 partially rescued GT outgrowth at E11.5. However, the GTs of *Shh* ^*CreERT2/+*^ *;p63* ^*−/−*^ *;Ctnnb1* ^*EX3/+*^ embryos exhibited severe abnormalities by E13.5. Lateral swelling was visible, but the GTs failed to develop at the mid‐region, showing a split shape with a hemorrhagic cloaca (Figure 4g).

## 4 DISCUSSION

### 4.1 Crosstalk between p63 and the Shh–Wnt/Ctnnb1–Fgf8 pathway during GT outgrowth

In this study, we elucidated the crosstalk between p63 and the Shh–Wnt/Ctnnb1–Fgf8 pathway during initial GT development. We demonstrated that *Shh* expression was not affected in *p63* KO embryos and that p63 was normally expressed in the cloaca of *Shh* KO embryos. Conversely, CTNNB1 expression was decreased in *p63* KO embryos, suggesting that p63 is required for CTNNB1 expression/activity independent of Shh signaling. p63α regulates CTNNB1 activity through phosphorylation of GSK3β, which regulates CTNNB1 stability (Patturajan et al., 2002). In either case, the loss of *Fgf8* expression in *p63* KO embryos may be caused by the decreased levels of CTNNB1 in the DUE. It has been reported that p63 could be directly regulated by Wnt/Ctnnb1 signaling through the binding of lymphoid enhancer binding factor 1 (LEF1) along with CTNNB1 (Chu et al., 2008; Ferretti et al., 2011); however, p63 was still expressed in GTs of the *Ctnnb1* conditional KO embryos used in this study.

The GT protrusion in *p63* KO mice was partly rescued by CTNNB1 activation, but the GTs of *Shh* ^*CreERT2/+*^ *;p63* ^*−/−*^ *;Ctnnb1* ^*EX3/+*^ embryos showed a split shape with a hemorrhagic cloaca and lacked a central structure by E13.5. *Shh* ^*CreERT2/+*^ *;Ctnnb1* ^*EX3/+*^ embryos (with wild‐type *p63* alleles) exhibited GT protrusion without such abnormal phenotypes, although they showed severe anorectal malformations (Miyagawa et al., 2014). p63 has diverse functions in a wide range of cellular processes, and plays an essential role in the cell fate commitment of the simple ectoderm to the epidermal lineage (McKeon, 2004; Mills et al., 1999; Wolff et al., 2009). GT development encompasses differentiation of ectodermal and endodermal stratified epithelia and indeed, the GTs of *p63* KO embryos resulted in a thinner cloacal membrane and undifferentiated GT ectoderm. It is therefore plausible that epithelial structures of the GT ectoderm, cloacal membrane, or both could not tolerate the rapid protrusion of buds elicited by CTNNB1 overexpression. Taken together, p63 is a key component of the signaling pathway that leads to *Fgf8* expression in the DUE and also contributes to GT outgrowth by maintaining structural integrity of the cloacal/GT ectodermal epithelia (Figure 4h).

### 4.2 Interaction of p63 and Ctnnb1, and *Fgf8* expression

In the current study, we found that p63 directly regulates *Fgf8* expression. p63 binds to CR3, a candidate limb and GT enhancer (Beermann et al., 2006; Miyagawa, Moon, et al., 2009), in nuclear extracts from GTs. Furthermore, this region conferred a transcriptional response to exogenous ΔNp63α, but not to TAp63, in luciferase assays. In *Shh* ^*CreER/+*^ *;Ctnnb1* ^*flox/−*^ embryos, *Fgf8* expression was reduced, but remained in the cloacal membrane (Miyagawa, Moon, et al., 2009). Thus, p63 may solely activate *Fgf8* expression in a *Ctnnb1*‐null background.

The current study showed that ΔNp63α is the predominant p63 isoform in GTs. The ΔNp63 isoform appears to be responsible for appendage formation because TAp63 isoform‐specific KO mice display no overt abnormalities and normal limb development (Guo et al., 2009; Su et al., 2009). The C‐terminal α/β domains of p63 are required for epidermal and limb development (Wolff et al., 2009). In addition, the α domain contains a functionally important SAM domain, which is essential for protein–protein interactions. One of the candidate interaction partners of the α domain is p300, and the p63α–p300 complex acts as a coactivator of Ctnnb1 (Katoh et al., 2019). p63 and CTNNB1 mutually activate the CR3 enhancer, but co‐expression of ΔNp63α and CTNNB1 did not show an augmented effect on CR3 enhancer activity in the current study. Therefore, the mode of interaction, if any, between p63 and CTNNB1 in developing GTs remains unclear.

The signaling network which elicits and maintains *Fgf8* expression might be more complex. p63 regulates the expression of a large number of genes, and some of them could cooperatively activate *Fgf8* gene expression. In the AER, *distal‐less homeobox 5*/*6* (*Dlx5*/*6*) genes are such candidates; *Dlx5*/*6* are downstream genes of ΔNp63 (Lo Iacono et al., 2008) and directly activate *Fgf8* expression (Restelli et al., 2014). Intriguingly, FGF8 counteracts peptidyl‐prolyl *cis*/*trans* isomerase (PIN1)‐mediated degradation of p63 protein, resulting in stabilization of ΔNp63 (Restelli et al., 2014). Thus, multiple genes/proteins participate in a regulatory loop that is essential for *Fgf8* expression in the AER. *Dlx5*/*6* are also expressed in the DUE, and *Dlx5*/*6* mutants show loss of urethral plate stratification, which is similar to *p63* KO phenotypes (Suzuki et al., 2008). Hence, the interaction of multiple factors including p63, DLX5/6, and CTNNB1 for regulating *Fgf8* expression in the DUE is complex and requires further investigation.

In the current study, we have extensively focused on *Fgf8* expression, but the outgrowth defects cannot be explained by a loss of *Fgf8* expression alone because the function of FGF8 is dispensable (Miyagawa, Moon, et al., 2009; Seifert et al., 2009). This is probably due to the compensation by other FGF ligands, which has been described in the limb, tooth, inner ear, pharynx, and brain (Boulet et al., 2004; Mariani et al., 2008; Moon & Capecchi, 2000; Watanabe et al., 2010). We have also shown that this compensatory mechanism occurs in the GT to maintain the amount of FGF signaling emanating from the DUE (Miyagawa, Moon, et al., 2009). The redundancy of FGF ligands was also analyzed in *Fgf receptor* (*Fgfr*) mutants and conditional *Fgfr1*/*2* double KO embryos, which revealed severe defects of GT outgrowth (Harada et al., 2015).

### 4.3 p63 and urogenital malformation

The initiation and outgrowth of GT primordia also critically influence anorectal/urogenital organ development, as revealed by the fact that perturbation of hedgehog and p63 signals results in severe anorectal/urogenital malformations (Cheng et al., 2006; Ince et al., 2002; Mo et al., 2001). Heterozygous *p63* mutations in humans are associated with several autosomal recessive congenital hereditary diseases, including ankyloblepharon‐ectodermal defects–cleft lip/palate syndrome (AEC; MIM 106260), ectrodactyly, ectodermal dysplasia, cleft lip/palate syndrome 3 (EEC3; MIM 604292), and split‐hand/foot malformation type 4 (SHFM4; MIM 605289). These disorders often comprise split‐hand/foot phenotypes or syndactyly of the hands and genital defects, such as hypospadias (Schmidt et al., 2022; Sutton et al., 2009). Hypospadias has been described as a malformation with a high prevalence and is accompanied by an ectopic orifice of the urethral meatus on the ventral side of the penis and ventral cleft of the urethral plate in severe cases. To our knowledge, expression of developmental regulatory genes associated with GT defects has not been reported in human *p63* mutation‐related diseases.

KO mouse studies have revealed that *Fgf8* expression in the DUE is required for both p63 and Shh signals. Each signal not only regulates CTNNB1 activity, but also elicits distinct gene expression; for example, p63 regulates *Bmp7* and *Dlx5*/*6* in the cloacal membrane (Suzuki et al., 2008). Conversely, Shh signal induces not only canonical Wnt ligands but also *Wnt5a*, *Bmp4*, and *Fgf10*, which are crucial regulators of GT development (Haraguchi et al., 2001; Perriton et al., 2002). Taken together, further study of the molecular mechanisms of genital organ development could shed light on the mechanisms underlying congenital abnormalities of the anorectal/urogenital organ systems.

## AUTHOR CONTRIBUTIONS

Shinichi Miyagawa and Gen Yamada conceived and designed the study. Shinichi Miyagawa, Daisuke Matsumaru, and Kentaro Suzuki conducted mouse preparation and sampling. Kosei Tanaka and Shinichi Miyagawa performed histological sectioning. Shinichi Miyagawa performed ChIP, luciferase, and immunoblotting assays. All authors investigated and discussed the morphology, histology, and gene expression results. Shinichi Miyagawa wrote the original draft, Kosei Tanaka and Shinichi Miyagawa prepared figures, and all authors reviewed, comment on, and edited the manuscript.
